# Genome-wide RNA pol II initiation and pausing in neural progenitors of the rat

**DOI:** 10.1186/s12864-019-5829-4

**Published:** 2019-06-11

**Authors:** Adam Scheidegger, Carissa J. Dunn, Ann Samarakkody, Nii Koney-Kwaku Koney, Danielle Perley, Ramendra N. Saha, Sergei Nechaev

**Affiliations:** 1 Department of Biomedical Sciences, University of North Dakota School of Medicine, Grand Forks, ND 58202 USA; 2 Present address: Omega Therapeutics, Cambridge, MA 02139 USA; 3 Molecular and Cell Biology Department, School of Natural Sciences, University of California Merced, Merced, CA 95343 USA; 4 Present address: Department of Pediatric Hematology-Oncology, Boston Children’s Hospital, Dana-Farber Cancer Institute, Harvard Medical School, Boston, MA 02115 USA

**Keywords:** RNA pol II, Transcription, Small RNA, Promoters

## Abstract

**Background:**

Global RNA sequencing technologies have revealed widespread RNA polymerase II (Pol II) transcription outside of gene promoters. Small 5′-capped RNA sequencing (Start-seq) originally developed for the detection of promoter-proximal Pol II pausing has helped improve annotation of Transcription Start Sites (TSSs) of genes as well as identification of non-genic regulatory elements. However, apart from the most well studied genomes of human and mouse, mammalian transcription has not been profiled with sufficiently high precision.

**Results:**

We prepared and sequenced Start-seq libraries from rat (*Rattus norgevicus*) primary neural progenitor cells. Over 48 million uniquely mappable reads from two independent biological replicates allowed us to define the TSSs of 7365 known genes in the rn6 genome, reannotating 2503 TSSs by more than 5 base pairs, characterize promoter-associated antisense transcription, and profile Pol II pausing. By combining TSS data with polyA-selected RNA sequencing, we also identified thousands of potential new genes producing stable RNA as well as non-genic transcripts representing possible regulatory elements.

**Conclusions:**

Our study has produced the first Start-seq dataset for the rat. Apart from profiling transcription initiation, our data reaffirm the prevalence of Pol II pausing across the rat genome and indicate conservation of pausing mechanisms across metazoan genomes. We suggest that pausing location, at least in mammals, is constrained by a distance from initiation of transcription, whether it occurs at or outside of a gene promoter. Abundant antisense transcription initiation around protein coding genes indicates that Pol II recruited to the vicinity of a promoter is distributed to available start sites of transcription at either DNA strand. Transcriptome profiling of neural progenitors presented here will facilitate further studies of other rat cell types as well as other organisms.

**Electronic supplementary material:**

The online version of this article (10.1186/s12864-019-5829-4) contains supplementary material, which is available to authorized users.

## Background

Transcription of genes was thought to be regulated mainly through recruitment of the RNA polymerase to promoters. However, work over the last several years [1–3] has demonstrated that mRNA production requires additional inputs even after the RNA polymerase has engaged a promoter and initiated RNA synthesis. Promoter-proximal Pol II pausing takes place within the first 100 nucleotides of many genes and, following a number of seminal studies (reviewed in [4, 5]), is now accepted as a common step in metazoan Pol II transcription. Regulated release of paused polymerase into productive transcription elongation accompanies key biological events including organism development and cellular responses to stimuli [2, 6–13]. Better understanding of transcription initiation, promoter-proximal pausing, and their contributions to transcription regulation is limited by lack of high-resolution datasets especially in commonly used model organisms like the rat. Discrepancies by a few nucleotides do not affect Chromatin Immunoprecipitation-sequencing (ChIP-seq) or mRNA-sequencing (RNA-seq) analyses because their effective resolution is relatively low. However, even single base pair inaccuracies impede analyses relying on the sequence context of promoters and other elements, including CRISPR/Cas9 based targeting [14]. With new technologies being rapidly developed, the demand for nucleotide-level precision of transcriptome annotations is only expected to increase. In addition to refining the Transcription Start Sites (TSSs) of known genes, there is also increasing interest in mapping non-genic transcription that does not produce stable RNA, but delineates non-genic regulatory elements [15–18].

Thus far, Pol II TSSs have been profiled with high depth and resolution only for relatively well-studied genomes of human, mouse, *C. elegans*, and *Drosophila* [18–22]. Here we turned to the rat, an important model organism that still has few available genome-wide datasets and, based on the experience with other genomes, is likely to have incomplete annotation of transcriptomic features. Small capped RNA sequencing, also referred to as Start-seq, captures short 5′-capped RNAs (TSS-RNAs) that are produced by Pol II during early transcription elongation [22, 23]. TSS-RNAs yield dual information: their 5′-ends precisely delineate the sites of transcription initiation, whereas their 3′-end positions indicate the locations of promoter-proximal pausing [22]. We report Start-seq in primary neural progenitors alongside poly-A selected high-coverage RNA-sequencing from the same RNA. We define high-confidence, base-pair resolution TSSs for 7365 of the ~ 24,000 currently annotated genes in the rn6 genome using the RefSeq annotation database, report the relationship of pausing with gene expression, and identify transcription start sites of new genes and potential non-genic regulatory elements. We identify general features of antisense transcription around gene promoters and characterize properties of Pol II pausing. The work outlines a high-resolution landscape of transcription initiation and Pol II pausing in rat neural progenitors of the rat and provides a workflow for transcriptional profiling of other cell types in the rat as well as in other organisms.

## Results

### Start-seq in rat neuronal progenitors

**Fig. 1:**
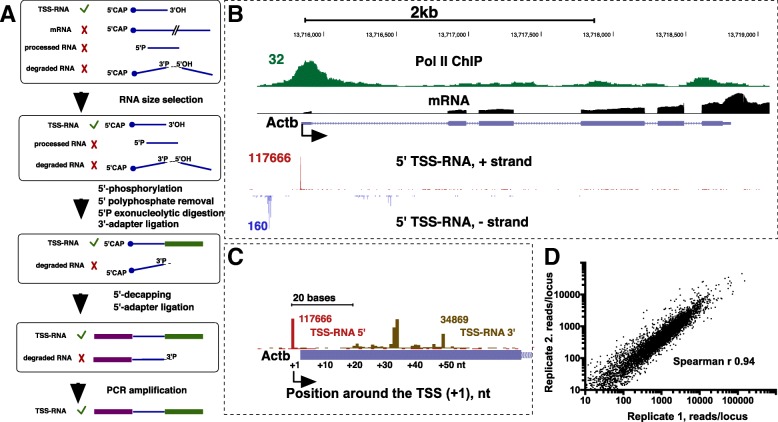
Validation of TSS-RNA sequencing in rat neural precursors. **a**. A scheme of an updated protocol for the preparation of Start-seq libraries for Illumina sequencing. **b**. UCSC browser shot of highly expressed *Actb* gene showing tracks from this study including 5′-tracks for TSS-RNA for each strand (red and blue) and mRNA-sequencing (black), alongside Pol II ChIP-seq (using an antibody against the Pol II N-terminus) track from mature rat neurons [10] representing a rat Pol II dataset that is most closely related to the current cell type. **c**. A zoomed-in view of *Actb* promoter-proximal region showing 5′- (red) and 3′-end (gold) of TSS-RNAs on this gene. The annotated *Actb* TSS is shown in blue bar and is located 2 bp downstream of the 5′ TSS-RNA peak. **d**. Correlation plot for promoter-proximal counts between two independent biological Start-Seq replicates. TSS-RNAs on the gene (sense) strand were counted in a +/− 500 bp interval from each RefSeq-annotated TSS

We isolated and sequenced small 5′-capped RNAs from neural progenitors of embryonic day 14 Sprague Dawley rats (see Materials and Methods). Preparation of Illumina Start-seq libraries is based on our earlier procedure that eliminates RNA species lacking the 5′-cap followed by preparation of small RNA libraries from the 5′-cap-enriched RNA pool [20–22] (Fig. 1a, Additional file 1: Figure S1, also see Methods). Compared with the published procedure, rather than excluding non-capped RNAs from ligation by treating with alkaline phosphatase, we directed these RNA species for degradation by adding a 5′-monophosphate with T4 polynucleotide kinase (PNK) prior to treatment with 5′-monophosphate-specific exonuclease (Fig. 1). The 3′-phosphatase-minus version of PNK was used to avoid removal of 3′-phosphates from RNA degradation products, which prevents their ligation to adapters and excludes them from libraries (Fig. 1a). For 5′-decapping, RppH enzyme (NEB) was used instead of the obsoleted Tobacco Acid Pyrophosphatase [19]. The resultant Start-seq procedure is shortened by at least 1 day by removing an extra gel size selection step, at which loss of RNA material commonly occurs [22].

Two independent biological replicates produced 27.9 M and 19.4 M Start-seq reads uniquely mappable to rn6 genome. Of these reads, 16,380,972 for replicate 1 and 7,395,642 for replicate 2 mapped within +/− 500 base pairs of annotated TSSs [24] of known genes. Selectivity of scRNAs for TSSs is also illustrated by examining individual genes. Even on a highly active Actin B (*Actb*) gene, a majority of Start-seq reads at *Actb* gene map within the gene promoter (Fig. 1b, c). When the numbers of TSS-RNA hits within +/− 500 bp from the same set of TSSs were compared, Spearman correlation between the replicates was 0.97 overall (Fig. 1d) and profiles of transcription initiation were very similar on individual genes (Fig. 1d, Additional file 1: Figure S2, and data not shown), attesting to consistency of the Start-seq procedure.

### Refinement of gene transcription start sites in the rat

**Fig. 2:**
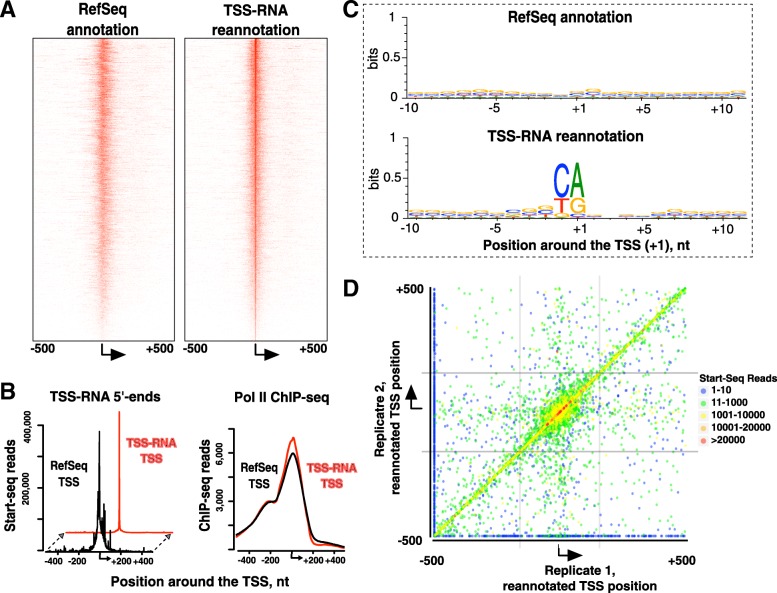
TSS RNA-based refinement of gene TSSs. **a**. Heatmaps of sense-strand TSS-RNA on 7112 rat genes ordered by decreasing TSS-RNA count within the promoter region, centered around annotated (left panel) and TSS-RNA peak-centered locations (right panel). **b**. Left pane. Metaplots of TSS-RNA 5′-ends for the same genes as in A, centered on TSSs defined using RefSeq (black) and TSS-RNA (red) TSS annotations. Right pane. Pol II ChIP-sequencing traces based on previously published data [10] centered against the same TSSs. **c**. Weblogo visualization of sequence enrichment of the genes centered around RefSeq annotation (top) and TSS-RNA reannotation (bottom panel). **d**. Correspondence between TSS annotations between two independent biological replicates, with each dot representing a gene and color coding indicating the number of TSS-RNA hits per gene as indicated. Genes are plotted against RefSeq-annotated TSSs. Strikethrough lines enclose genes considered for TSS reannotations based on each replicate, with the center quadrant containing genes that were reannotated

As the 5′-ends of TSS-RNAs pinpoint the sites of transcription initiation [18, 20, 22], we compared TSSs of mRNA genes defined with Start-seq to the current (rn6) rat gene annotations. To identify genes on which we could annotate TSSs, we first discarded genes with fewer than 10 TSS-RNA sense strand reads in either replicate as noise. To avoid impinging on neighboring transcripts, we also included a distance filter for maximum distance to RefSeq TSS (Fig. 2d, Additional file 1: Figure S3). Out of the 9158 rn6-annotated genes with TSS-RNA signal above the noise threshold, 7365 met our location criteria and 7112 of these genes had unique gene ids. Reannotated candidate TSSs locations were exactly identical for 4730 genes and were within +/− 10 nt of each other for 5508 genes. Among the qualifying genes, we determined the nucleotide position with the largest number of 5′-ends of reads mapped to the sense strand. Only 1842 genes showed Start-seq TSS locations within +/− 5 nt of the annotated TSS. There was no bias toward upstream versus downstream shift of RefSeq versus Start-seq TSSs, suggesting that no single mechanism accounts for these differences (Fig. 2d and Additional file 1: Figure S3). There was overall sharpening of TSS-RNA distributions when arranged against their peak positions instead of RefSeq TSSs (Fig. 2a, b, left pane).

To validate the TSS reannotations, we compared the DNA sequence context around the observed Start-seq gene TSSs to those around existing TSSs in rn6 genome. While RefSeq positions showed no DNA sequence enrichment, following reannotations (Fig. 2), a clear Pol II initiator (Inr) sequence motif [25] centered around TSS-RNA defined locations was observed (Fig. 2c), similar to what was previously found in human and mouse datasets [20, 21]. Apart from validating the TSS annotations, the data also reaffirm conservation of Pol II initiation sequence context in mammals. To verify the sensitivity of Start-seq based TSS annotations, especially at lower TSS-RNA coverage loci, we divided the 7365 TSSs into quartiles based on the number of mapped TSS-RNA reads. Enrichment of the Inr motif persists even at low (between 10 and 300 reads per 1000 bp TSS window, Additional file 1: Figure S4) coverage, indicating that our Start-seq noise threshold is conservative. In contrast, the existing rn6 RefSeq annotations do not contain sequence motif information even for the most highly expressed gene quartile (Additional file 1: Figure S4). To avoid a potential bias of RNA-based readout datasets, we also utilized previously published RNA Pol II ChIP-seq data, obtained from mature rat neurons (GSM565202) [10]. We observed sharpening of Pol II ChIP-seq metagene signal (Fig. 2b, right pane), suggesting that TSS-RNA method of reannotating start sites is not biased for a specific technique. Reannotated TSSs are listed in Additional file 2.

### Antisense transcription around mRNA genes

**Fig. 3:**
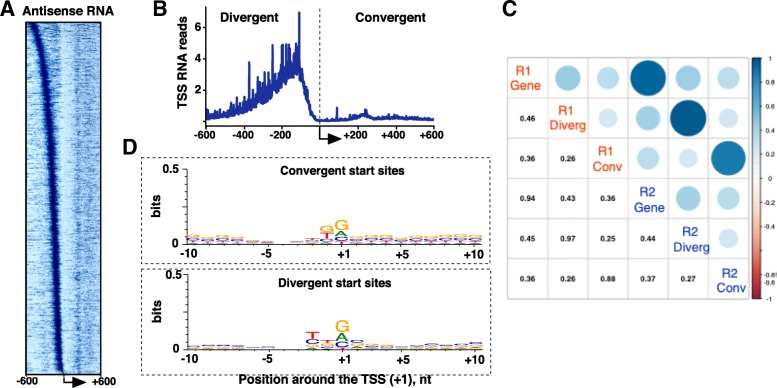
Antisense transcription in the rat genome. **a**. A heatmap representing antisense transcription sorted by the distance of divergent peak from the gene TSS, indicated by arrow. Of the 7112 genes, 601 genes that did not contain divergent signal within 600 bp from the TSS were removed from the heatmap. Due to low signal from antisense, and especially convergent transcription, the image was enhanced with Pixelmator to highlight convergent transcription. **b**. Metagene plot of antisense transcription relative to the reannotated gene TSSs. **c**. Spearman r correlation matrix of sense and antisense transcription, per replicate. Counts for convergent and divergent transcription were defined on the appropriate strand within +/− 50 nt from the location with the highest signal. **d**. Weblogo representation of DNA sequence context centered around Convergent (top) and Divergent (bottom) peak locations

Transcription initiated from the strand opposite to the promoter has been described around human and mouse genes [26–30], but its distribution in the rat genome remains uncharacterized. The so-called divergent transcription starts upstream of the promoter and is directed away from it [26, 27, 29]. We defined the divergent TSS for each gene as the base pair with the most Start-seq hits on the strand opposite to the gene within a 500-bp interval upstream of the promoter. TSS-RNAs in rat neuronal progenitors indicate prevalent divergent transcription initiation (Fig. 3a, b). While there is no set distance between sense and divergent start sites for genes across the genome - similar to the mouse data [20] - a majority of divergent peaks are found ~ 80–200 nt upstream of the gene TSS (Fig. 3a, b), with good correlation of divergent transcription signal magnitude between individual Start-seq replicates (Fig. 3c). Locations of the highest peaks of divergent transcription were less consistent (Fig. 3a), indicating the limiting sequencing coverage for these events or, more likely, intrinsically lower precision of divergent transcription initiation.

Convergent transcription initiates downstream of the TSS and is directed head-on into the promoter. After applying the same 10-count TSS-RNA noise threshold, we detected convergent transcription on 2531 genes within a 500-bp window downstream of the main promoter (Fig. 3b). Because convergent transcription is even lower in intensity than divergent, this threshold under-reports transcription at current sequencing coverage. The site of convergent initiation was defined similar to the divergent TSS, that is, using the base pair position with the highest signal downstream of the main TSS. The convergent signal was lower than that from divergent initiation, and convergent initiation sites were also concentrated further away, ~ 200–250 nt downstream of the sense TSS than divergent transcription.

Just like the gene TSSs, divergent and convergent TSSs are enriched with an Inr-like sequence motif (Fig. 3d), indicating a common mechanism of Pol II initiation both at and outside of gene promoters. There is a modest yet positive correlation between the magnitude of gene TSS-RNA signal and its associated antisense, both divergent and convergent, transcription initiation signal ((Spearman, 0.36–0.46) Fig. 3c). Taken together, these data reinforce the notion that antisense Pol II initiation is common throughout mammalian transcription [26–31] and may be co-regulated with the main promoter.

### Promoter-proximal pol II pausing is ubiquitous across the rat genome

**Fig. 4:**
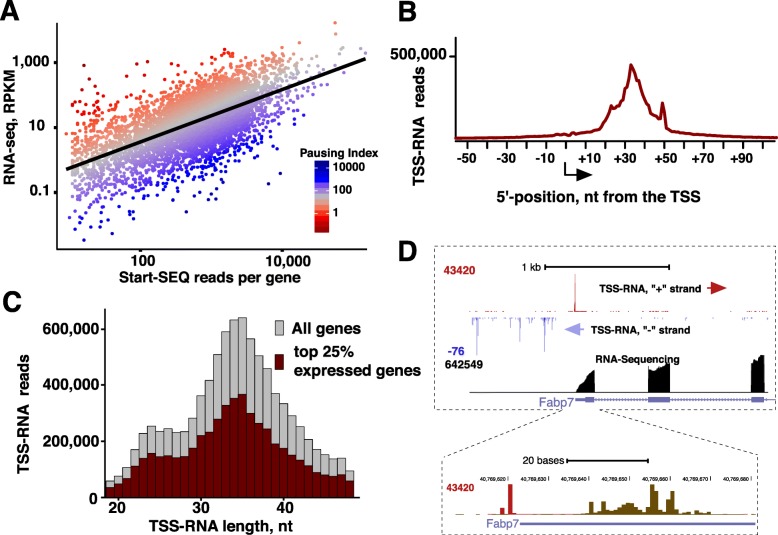
Pol II pausing across the rat genome. **a**. Scatter plot showing the distribution of TSS-RNA versus RNA-seq FPKM signal for all genes, with pausing index indicated by color. **c**. Metaplot of TSS-RNA 3′-ends relative to the reannotated TSS. **b**. Metaplot of TSS-RNA lengths around the TSSs. All RNAs on the sense strand from a gene within +/− 150 bp interval were considered regardless of their initiation site. All genes versus top 25% highest expressed genes (based on RNA-seq signal FPKM) are shown, respectively, in grey and dark red. **d**. A UCSC browser shot showing Pol II pausing and transcription on *Fabp7* gene, which has the highest expression in rat neural progenitors based on RNA-seq analysis. Inset shows a zoomed-in view of the gene’s promoter region

While TSS-RNAs are generated by Pol II pausing, their levels on a gene are determined through dynamic interplay between mechanisms that establish pausing and those that release paused Pol II into elongation (reviewed in [4, 32, 33]). To quantify pausing, TSS RNA signal is commonly normalized for gene expression by calculating the Pausing index (PI), also referred-to as traveling ratio [2, 6, 26]. RNA-sequencing is the best currently available gene expression measurement for these cells and has been used before to estimate PI [18, 22]. Comparing PI against mRNA levels of the same gene reaffirms positive but modest (0.60, Spearman, Fig. 4a) correlation of PI with gene expression. Gene Ontology (GO) enrichment analysis indicated that, consistent with earlier work in *Drosophila* and mouse [20, 22], that higher PI tends to favor genes involved in development and stimulus response, whereas low PI genes are skewed toward metabolism (Additional file 3). We did not detect statistically significant enrichment of cell lineage specific genes related to neural development. Thus, pausing likely represents an intrinsic, rather than conditional, property of genes [34, 35].

As the 3′-ends of TSS-RNAs define the locations of Pol II pausing [22], we next determined the positions of TSS-RNA 3′-ends. Metagene analysis around the TSSs shows that TSS-RNA 3′-ends peak around + 35 nt downstream of the TSS (Fig. 4b, Additional file 1: Figure S6). This is similar to our previously defined distribution of TSS-RNAs in other organisms, including *Drosophila*, human, and mouse [20–22], pointing to commonality of mechanisms that establish Pol II pausing across metazoans.

Ever since the original discovery of promoter-proximal Pol II pausing [36–39], there remains a question about pervasiveness of pausing and, particularly, existence of non-paused genes (for example, [2, 7, 8, 12, 40]). In genome-wide datasets, paused genes are normally defined through threshold-based cutoffs in global PI distribution [1, 26, 35], which under-reports paused and over-represent non-paused genes. Even then, a majority of genes have Pol II accumulation at promoters indicative of pausing [3]. Apart from completely inactive genes, low PI values should stem from active genes. However, these genes still show detectable Start-seq signal and have the same RNA size distribution as the rest of the genes, both overall (Fig. 4c) and on a representative gene with a high expression level based on RNA-seq signal (Fig. 4d). Examining individual genes, we failed to find an active gene without TSS-RNA signal (data not shown). While quantitative differences probably reflect genome-specified differential duration of premature transcription termination, presence of scRNA at the right location is detected on all active genes we examined. These observations indicate that Pol II pausing occurs on most if not all genes and that there would be few, if any, “nonpaused” active genes, at least in steady-state cells.

### Pol II pausing is globally constrained by distance from transcription initiation

**Fig. 5:**
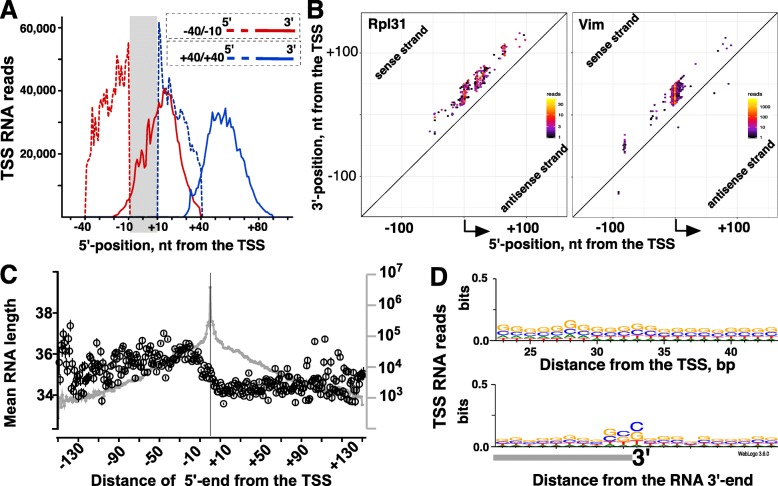
Pol II pausing and distance from transcription initiation. **a**. Metaplot of TSS-RNAs initiating outside of the exact TSS region +/− 10 bp (greyed out), which was excluded from this analysis. The 5′-ends of RNAs are shown in dashed lines and their 3′-ends in solid lines. RNA initiating at more upstream regions are shown in red and at downstream regions in blue. **b**. 2-d scatter plot of initiation on two genes with top 20 FPKM values as in [31]. **c**. TSS-RNA length versus their initiation location around the TSS. Circles show mean (with SEM) for TSS-RNA lengths metaplot for RNAs initiating at the indicated locations (positions) relative to the TSS (vertical line). Grey line (right Y-axis) shows the number of RNAs mapping (metagene) to each location in Replicate 1. **d**. Weblogo plot of DNA sequence context around locations of Pol II pausing based on distance from the reannotated TSS (top) and relative to 3′-end of each RNA

Two mechanisms can, in principle, define the location of Pol II pausing: the DNA sequence context or distance from transcription initiation [41–43]. To determine the potential contributions of each mechanism, we focused on initiation events that occur just outside of gene TSSs in the intervals between -10 to -5 nt from the peak TSS (upstream interval) and +5 to +40 nt from the peak TSS (downstream interval). If pausing is driven entirely by the underlying DNA sequence, then pausing should happen at the same location irrespective of the initiation position. TSS-RNAs should thus be longer for – in reference to the TSS – upstream and shorter for downstream initiation events. In contrast, sequence-independent mechanisms would result in similar RNA lengths regardless of the initiation site and accordingly shifted positions of 3′-RNA ends. TSS-RNAs initiated immediately upstream of the TSSs in a metagene plot show that the 3′-ends of upstream-initiating reads are shifted upstream, and downstream-initiating reads have 3′-ends shifted accordingly downstream of the events initiating precisely at the TSS (Fig. 5a). Examination of individual genes (Fig. 5b and data not shown) also points to upstream-initiating reads ending at more upstream locations, indicating that pausing for upstream-initiating events is shifted upstream accordingly. These observations argue for a major contribution of sequence-agnostic mechanisms to defining pausing location across the genome.

Analysis of RNA lengths relative to each RNA 5′-end rather than the gene TSS [31] shows a distribution of lengths peaking around ~ 35 nucleotides, similar to TSS-centric analysis. However, we noted that upstream initiated RNAs are, on average, 2–3 nucleotides longer than RNAs initiating downstream of the TSS. The TSS itself appears to be the inflection point (Fig. 5c and Additional file 1: Figure S5). While the reasons for this difference remain to be investigated, we suggest that this may be due to different availability, or activity, of factors such as NELF or TFIIS for initiation at different locations. Notably, this extra length does not compensate for the additional distance upstream from the TSS, retaining the RNA length constraints. Analysis of the sequence context around the RNA 3′-ends (but not distance from the TSS) shows preference for G/C nucleotides (as also determined recently [31]), indicating that generation of TSS-RNAs (either the initial pausing or subsequent Pol II backtracking) does to some extent depend on the sequence context (Fig. 5d) [43]. Initiation events outside of promoters, namely, divergent transcription, where sequences are not likely to have specifically evolved to control transcription, had similar distributions of RNA sizes (Additional file 1: Figure S6). Taken together, these data indicate that pausing is a common, likely requisite step of Pol II transcription regardless of whether it initiates at or outside of gene promoters [30]. The data also suggest that the location of pausing, while to some extent sensitive to the sequence context, is ultimately constrained by a distance from transcription initiation.

### New transcription initiation elements identified from transcriptome sequencing

**Fig. 6:**
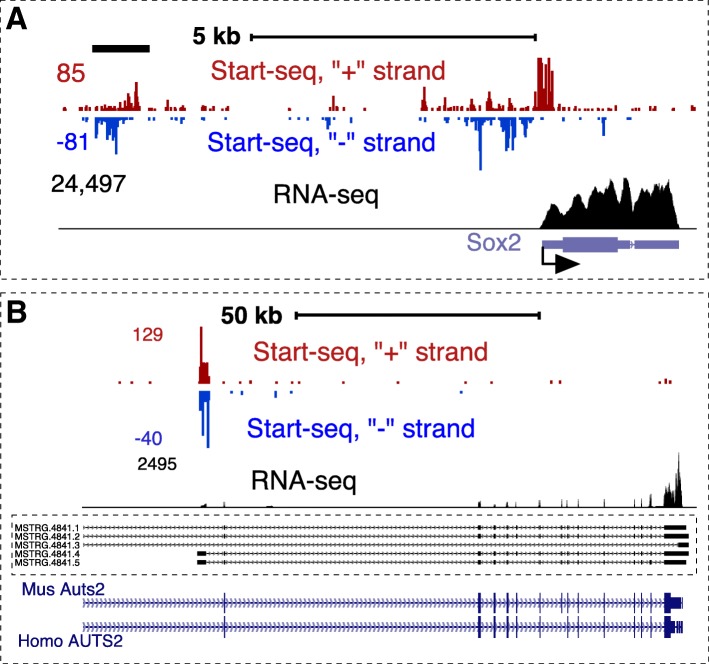
Examples of transcriptome elements identified in the rat genome. **a**. A potential regulatory element (enhancer) upstream of *Sox2* gene defined based on bidirectional TSS-RNA signal and low RNA-seq signal. **b**. Example of a new annotation in the rat genome showing homology to *Auts2* gene. Transcripts assembled from RNA-seq data are shown inside a bracket. Mouse and *H. sapiens* genes are shown underneath

Transcription initiation events can indicate genes producing stable RNA or non-genic regulatory elements such as transcriptional enhancers. By combining Start-seq with polyA RNA-sequencing, we sought to identify transcription initiation events that are not annotated in the databases [18, 20]. To identify new genes, we used our RNA seq dataset for transcript assembly with *stringtie* [44]. We identified 100% (17,175) of the known genes and 100% of the exons in the reference annotation, suggesting that the RNA-seq coverage is sufficiently high for annotation of gene transcripts. In addition, *stringtie* identified 6219 novel intergenic transcripts with 4651 (74.7%) of those containing more than one exon. These transcripts represent potentially novel genes in the rat genome (Additional file 4). Because a combination of HISAT2 and *stringtie* can over-report single exon transcripts [45], we considered new genes only among multi-exon transcripts, even at a cost of under-representing bona-fide single-exon genes. Figure 6b shows one such gene with a typical assembled RNA transcript and Start-seq signal at the would-be promoter location. This transcript is homologous to the AUTS2 locus in the mouse and human, a gene implicated in neurodevelopment processes [46] (Fig. 6b). The mean RNA expression of new genes is ~ 2-fold lower, but the overall distribution of expression levels is compatible to expression of known genes (Additional file 1: Figure S7), suggesting that low level expression is not the main reason for incomplete annotations and pointing that exploration of transcriptome in other cell types is worthwhile. Start-seq coverage of these genes was accordingly lower than that of the known genes (Additional file 1: Figure S7).

To identify potential regulatory elements, we used Homer [47] to find Start-seq peaks on both strands across the genome after excluding the known genic start sites (+/− 3 kb from the gene’s start site) found in either the Ref-Seq or our assembly. This resulted in 29,481 homer peaks. Because accessible genomic elements of including transcriptional enhancers are characterized by bidirectional transcription [20, 48–50], we used these peaks to identify regions of bidirectional TSS signal enrichment (Additional file 5, see Methods). Figure 6a shows one of those regions approximately 7 kb upstream of the Sox2 gene. This region represents an active enhancer near a transcriptionally productive developmental gene in these neural progenitor cells. The identified new genes and non-genic TSSs are listed in the Additional file 1.

## Discussion

Using small capped RNA sequencing (Start-seq), we profiled Pol II transcription start sites and pausing in neural progenitors of the rat. Compared with human and mouse, the rat genome appears to be even more misannotated for gene TSSs and likely other genomic elements as well. By refining TSSs of known genes and identifying thousands of new TSSs of potential genes and non-genic elements, the first Start-seq datasets in the rat reported here will facilitate transcriptome profiling in other cell types of the rat as well as other organisms. Our definitions of new genes and non-genic elements are likely conservative, so that additional datasets are expected to further improve the scope and confidence of rat transcriptome annotations in various cell types.

Nascent RNA analysis methods such as Global Run-On and Precision Run-On sequencing (GRO-seq and PRO-seq) are powerful tools for transcriptome profiling [19, 26]. Start-seq is not able to measure expression of genes, but unlike the Run-On methods, it can profile transcription initiation events in specimens with inactivated RNA polymerase. Broader adoption of Start-seq has been limited by technical complexity. We have streamlined the Start-seq method by reducing the bench time it takes to complete the protocol. Future iterations of Start-seq method development will increase the specificity of 5′-capped RNA recovery and reduce the requirement for starting RNA material. For example, ribosomal RNAs constituted 15.7 and 44.9% of reads, respectively, in each replicate; the difference in the abundance of TSS-RNAs across replicates is consistent with the relative abundance of ribosomal RNA reads in each sample, indicating that rRNA reads constitute a major variable in Start-seq libraries, presumably at the level of RNA size selection during library preparation (Additional file 1: Figure S1). Combining Start-seq with rRNA depletion, as was recently done in *Drosophila* [18], should help circumvent this issue. Given the growing affordability of sequencing, it may also be prudent to opt for a higher sequencing depth instead of extra steps in Start-seq library preparation.

Start-seq allowed us to visualize the overall Pol II initiation landscape across the rat genome. We reaffirmed the prevalence of convergent and divergent initiation around Pol II-transcribed genes in the rat. The distances of antisense initiation sites to main TSSs vary widely among genes and, therefore, rather than specific sequences, are likely defined by topological features of the genome such as chromatin looping and/or sequence features such as CpG islands. Convergent initiation in general is shifted further away from the main TSS than divergent initiation, probably because the former takes place next to the + 1 nucleosome [35, 51, 52]. The magnitudes of sense and antisense signals show modest but positive correlation with transcription of the gene, which is comparable to correlation between pausing and gene expression, indicating that these events are co-regulated. We suggest that the Pol II machinery is commonly brought to the vicinity of the promoter (or a transcription factory [53, 54]) and then distributed according to its affinity to each potential start site within the local environment.

Pol II pausing involves a complex interplay of processes that include RNA capping, initial pausing, backtracking, and premature termination (reviewed in [31, 33, 55]). The location of pausing on genes in relation to the start site appears to be highly conserved across metazoans and peaks around ~ 35 nt from a gene TSS. Using Start-seq, we did not detect the bimodal distribution of TSS-RNA lengths observed in PRO-seq based experiments [31], consistent with earlier TSS-RNA data in mammalian organisms [20–22], although individual genes such as *Actb* do show that (Figs. 1b,c and 5b). This may be because TSS-RNA and PRO-seq detect nonidentical populations of RNA generated at different stages of Pol II pausing including processing and backtracking. Because pausing appears to take place during transcription at and outside of promoters [30], likely through the same underlying mechanisms, pausing may be better termed as initiation-proximal rather than promoter-proximal pausing.

Contribution of sequence-dependent and sequence-independent mechanisms to the establishment of Pol II pausing and subsequent Pol II release remain to be fully understood. We suggest that positioning of Pol II from the TSS determines where promoter-proximal pausing would occur. Conservation of pausing among different organisms and at sequence contexts throughout the genome, at and outside of gene promoters, indicates sequence-independent, likely universal mechanisms. Pausing establishing factor NELF (Negative Elongation Factor) [56, 57] and DRB Sensitivity Inducing Factor (DSIF) govern pausing on most, if not all, transcription events [58–60]. For example, NELF, through its multiple RNA binding sites [61, 62] or DSIF [59], may serve as a “ruler” to measure the distance of initial pausing or to define the location of subsequent backtracking. Given that pausing is also constrained by the sequence context [43], at least within up to five nucleotides, multiple mechanisms are likely at play. We suggest that the length-based universal constraints define the upper limit for pausing whereas DNA sequence, or balance of promoter activity and pause release, can alter that within these limitations [63]. Indeed, locations of 3′ ends vary on individual genes from 25 to 50 + nt (Figs. 4 and 5 and data not shown). Small RNAs reflect the complex processes during Pol II pausing and release [64], and their analysis in different systems and under different conditions will help shed light on these mechanisms.

By combining Start-seq and RNA-seq data from the same cells, we performed an initial profiling of genic and non-genic TSSs of the rat. This approach can be used for other systems, especially to map the noncoding transcription landscape. While our RNA-seq data detected 100% of known rn6 mRNAs and 100% of known exons, we are unlikely to have fully saturated the rat transcriptome by analyzing one cell type because some genes have low activity in these cells, especially for noncoding transcripts. The number of identified non-genic elements based on TSS-RNA in the rat is on a lower side of the numbers of enhancers reported based on histone marks [65–67]. Future analyses of RNA datasets will advance transcriptome annotations in various cell types of the rat as well as other, less studied organisms.

## Conclusions

Applying an improved Start-seq procedure for rat neuronal progenitors and combining it with polyA RNA-sequencing from the same sample sets, we report the transcription initiation landscape in these cells that includes (i) refinement of known gene transcription start sites; (ii) profiling of antisense (divergent and convergent) transcription initiation; (iii) genome-wide profiling of Pol II pausing at and outside of gene promoters and (iv) identification of new genes and potential regulatory elements. The work presented here will help fine-tune DNA sequence-based approaches (e.g., CRISPR targeting) in rats and facilitate transcriptome profiling of other rat cell types as well as analyses of other organisms.

## Methods

### Animals and derivation of neuronal progenitors

All animal procedures were performed in accordance with the National Institute of Environmental Health Sciences (NIEHS) and the University of California Merced animal care committee’s regulations [NIEHS Institutional Animal Care and Use Committee (IACUC) approval: ASP#01–21; and University of California Merced IACUC approval: ASP#13–0007 and ASP#16–0004]. Time-pregnant rats were obtained from a commercial resource (Charles River). Pregnant dams were sacrificed by first deeply anesthetizing them (to minimize pain sensation during decapitation) by intraperitoneal injection of pentobarbital solution and then decapitated using a sharp guillotine. Embryonic animals were decapitated with a sharp pair of scissors, cortices were isolated and subsequently digested in Accutase (Gibco) for 5 min at room temperature. Cultures of cortical neural progenitors were prepared from embryonic day 14 (E14) Sprague Dawley rats of either sex. Single cell suspension was achieved by triturating digested tissue through fire-polished Pasteur pipettes. Cells were washed with Hank’s Balanced Salt Solution (HBSS) without calcium and magnesium, and then plated onto dishes coated with CELLstart (Gibco) in Knockout DMEM/F-12 (Gibco) supplemented with 2% StemPro Neural Supplement (Gibco), 2 mM GlutaMAX (Gibco), 20 ng/ml bFGF (Gibco), and 20 ng/ml EGF (Gibco). Cells were passaged every 2–3 days and routinely collected after the second passage.

### Total RNA preparation

PolyA selected RNA libraries were prepared from 50 ng of total RNA extracted from frozen cell pellets using Trizol reagent. In addition to the standard Trizol procedure, we included a chloroform extraction step of the aqueous phase after chloroform-induced separation of phases, to remove traces of phenol. RNA Integrity Numbers were calculated by Bioanalyzer and were always > 6.8, to meet the RNA quality guidelines for RNA sequencing service (Novogene). PolyA-selected RNA-sequencing libraries were prepared using NEBNext Ultra II library preparation kit with NEB beads, using 12 cycles of amplification. Libraries were quantified on Bioanalyzer prior to sequencing.

### Start-seq library preparation

scRNAs were prepared based on our earlier procedure [21, 22], with modifications. In brief (5*10^7) cells were used to extract nuclei by washing with hypotonic lysis buffer [22] followed by preparation of total RNA using Trizol reagent, size selection on 15% Urea-TBE gel (Novex), and crush and soak elution using cellulose acetate spin filters (Agilent cat# 5185-5990). After ethanol precipitation, size selected RNA was treated, successively, with T4 Polynucleotide Kinase 3′ phosphatase minus (New England Biolabs, NEB), 5′-polyphosphatase, terminator exonuclease (both Epicentre), followed by ligation of 3′-Illumina small RNA Tru-Seq adapter using T4 RNA Ligase 2, truncated K225Q (NEB). Reactions were then purified on 15% Urea-TBE gel (Novex) to select 45-100 nt RNA sizes, extracted from the gel as above and treated with Rpph (NEB) in Thermopol reaction buffer. After ligating the Illumina 5′-Tru-Seq small RNA adapter with WT T4 ssRNA Ligase 1 (NEB) in the presence of ATP, reverse transcription was done per Tru-Seq Illumina Small RNA kit and libraries were amplified for 18 PCR cycles. Phenol-chloroform, chloroform, and ethanol precipitation was used between each enzymatic treatment. PCR-amplified libraries were purified on a 6% TBE gel to remove linker dimers, extracted from the gel as above, and quantified using Bioanalyzer (Agilent) and droplet digital PCR (Bio-Rad) prior to sequencing.

### Sequencing and initial data processing

Start-Seq libraries were sequenced on a MiSeq instrument for quality control locally and re-sequenced on HiSeq2500 using small RNA option (SE50) commercially (Novogene) to the depth of ~ 100 M raw reads per sample. Raw files for Start-seq were mapped to rn6 genome using Hisat2. To filter out highly abundant species with multiple genomic copies such as tRNAs, only uniquely mappable Start-seq reads (Hisat2 mapping score > 3) were considered for analysis. Mapped reads were assigned to annotated genes using rn6 RefSeq annotation.

### RNA-sequencing analysis

PolyA-selective RNA sequencing was done to an average ~ 140 M raw reads per replicate using a commercial company (Novogene) from Trizol-extracted RNA. Reads were aligned to the rn6 genome using STAR and expression levels (FPKM) were obtained using the Rsubread [68] and DESeq2 [69] R packages. Transcripts were assembled using stringtie with default parameters.

### Small RNA data analysis

Rn6 annotated gene TSS locations were obtained from UCSC and deduplicated to produce a list of unique start site coordinates for each gene. Contaminating RNAs (tRNA, rRNA, etc) and micro RNA species were removed from consideration for this study. The deeptools package [70] was used to convert alignment files to bigwig (bamCoverage) and to count reads +/− 500 base pairs around the TSS locations defined previously (computeMatrix). Strand information was preserved, and reads were counted in the sense direction for all genes both on the 5’ and 3’ of the reads. After fitting the location of the highest peak in each (annotated TSS-centered) gene window to normal distribution, the range of 1 SD from the mean (Additional file 1: Figure S1) (146 nt for replicate 1 and 149 nt for replicate 2 (Additional file 1: Figure S3) was used as the maximum distance to define genes on which we could reannotate TSSs. Custom R scripts were used to analyze transcription around these sites. Pausing Index (PI) was calculated as the ratio of scRNA signal within the TSS +/− 500 bp window in the sense direction and RNA-seq-derived expression level (FPKM) of the same gene. Metagene plots and heatmaps were made using MakeHeatmap or custom R scripts. Due to sequencing read length of 50, the maximum length insert we could identify by adapter trimming was 47, and therefore sequences longer than 48 nt are not represented in RNA length-based analyses, although lower-coverage paired end sequencing of the same Start-Seq libraries (Supplement) shows that the size distribution calculated from paired end read sequencing is the same. Individual Start-seq replicates were processed independently and, unless indicated otherwise, replicate 1, which contained higher coverage, is shown in main Fig. 2-d plots for Start-seq RNA were made with the R package ggplot using coordinates of individual genes relative to their TSS-RNA reannotated TSSs. For identification of new TSS elements, peaks called by Homer using “factor” and “separate strand” flags were filtered to exclude peaks inside all gene promoter regions (+/− 3 kb from each promoter) using annotatePeaks from the same package. To identify bidirectional regions of TSS-RNA enrichment, peaks called by homer were filtered to exclude those near gene start sites (+/−3kbp from each TSS). Among the remaining peaks, those that were within 3000 bp from each other were merged using bedtools if at least two of the adjacent peaks were on opposite strands. This resulted in ~ 8600 bidirectional TSS regions.

## Additional files

Additional file 1: Supplemental figures and supplemental figure legends. (DOCX 1323 kb)

Additional file 2: The list of genes with rn6-based coordinates of TSSs (RefSeq and Start-Seq-based) alongside TSS-RNA signal and RNA-seq signal for each replicate. (XLSX 1592 kb)

Additional file 3: Panther GO analysis of highest and lowest PI genes. (XLSX 19 kb)

Additional file 4: Stringtie assembly of new transcripts, as a Gene Transfer Format (GTF) file. (TXT 11349 kb)

Additional file 5: Regions of bidirectional transcription outside of known genes identified based on TSS-RNA. (TXT 211 kb)

## Data Availability

Data generated in this work are deposited to the GEO database under accession # GSE130338. ChIP-sequencing data analyzed here are from GEO accession # GSM565202.

## Abbreviations

bFGF: basic fibroblast growth factor
bp: base pairs
ChIP-seq: Chromatin Immunoprecipitation Sequencing
DRB: 5,6-Dichlorobenzimidazole 1-β-D-ribofuranoside
DSIF: DRB Sensitivity-Inducing Factor
EGF: Epidermal growth factor
NEB: New England Biolabs
NELF: Negative ELongation Factor
nt: nucleotides
PI: Pausing index
PNK: Polynucleotide kinase
Pol II: RNA polymerase II
PRO-seq: Precision Run-On Sequencing
TSS: Transcription Start Site

## References

[1] Kim TH, Barrera LO, Zheng M, Qu C, Singer MA, Richmond TA, Wu Y, Green RD, Ren B (2005). A high-resolution map of active promoters in the human genome. Nature.

[2] Muse GW, Gilchrist DA, Nechaev S, Shah R, Parker JS, Grissom SF, Zeitlinger J, Adelman K (2007). RNA polymerase is poised for activation across the genome. Nat Genet.

[3] Core LJ, Waterfall JJ, Gilchrist DA, Fargo DC, Kwak H, Adelman K, Lis JT (2012). Defining the status of RNA polymerase at promoters. Cell Rep.

[4] Adelman K, Lis JT (2012). Promoter-proximal pausing of RNA polymerase II: emerging roles in metazoans. Nat Rev Genet.

[5] Venkatesh S, Workman JL (2015). Histone exchange, chromatin structure and the regulation of transcription. Nat Rev Mol Cell Biol.

[6] Zeitlinger J, Stark A, Kellis M, Hong JW, Nechaev S, Adelman K, Levine M, Young RA (2007). RNA polymerase stalling at developmental control genes in the Drosophila melanogaster embryo. Nat Genet.

[7] Boettiger AN, Levine M (2009). Synchronous and stochastic patterns of gene activation in the Drosophila embryo. Science.

[8] Min IM, Waterfall JJ, Core LJ, Munroe RJ, Schimenti J, Lis JT (2011). Regulating RNA polymerase pausing and transcription elongation in embryonic stem cells. Genes Dev.

[9] Gilchrist DA, Fromm G, dos Santos G, Pham LN, McDaniel IE, Burkholder A, Fargo DC, Adelman K (2012). Regulating the regulators: the pervasive effects of pol II pausing on stimulus-responsive gene networks. Genes Dev.

[10] Saha RN, Wissink EM, Bailey ER, Zhao M, Fargo DC, Hwang JY, Daigle KR, Fenn JD, Adelman K, Dudek SM (2011). Rapid activity-induced transcription of arc and other IEGs relies on poised RNA polymerase II. Nat Neurosci.

[11] Chen K, Johnston J, Shao W, Meier S, Staber C, Zeitlinger J (2013). A global change in RNA polymerase II pausing during the Drosophila midblastula transition. Elife.

[12] Lagha M, Bothma JP, Esposito E, Ng S, Stefanik L, Tsui C, Johnston J, Chen K, Gilmour DS, Zeitlinger J, Levine MS (2013). Paused pol II coordinates tissue morphogenesis in the Drosophila embryo. Cell.

[13] Williams LH, Fromm G, Gokey NG, Henriques T, Muse GW, Burkholder A, Fargo DC, Hu G, Adelman K (2015). Pausing of RNA polymerase II regulates mammalian developmental potential through control of signaling networks. Mol Cell.

[14] Sanson KR, Hanna RE, Hegde M, Donovan KF, Strand C, Sullender ME, Vaimberg EW, Goodale A, Root DE, Piccioni F, Doench JG (2018). Optimized libraries for CRISPR-Cas9 genetic screens with multiple modalities. Nat Commun.

[15] PC ENCODE (2012). An integrated encyclopedia of DNA elements in the human genome. Nature.

[16] Hnisz D, Abraham BJ, Lee TI, Lau A, Saint-André V, Sigova AA, Hoke HA, Young RA (2013). Super-enhancers in the control of cell identity and disease. Cell.

[17] Franco HL, Nagari A, Malladi VS, Li W, Xi Y, Richardson D, Allton KL, Tanaka K, Li J, Murakami S, Keyomarsi K, Bedford MT, Shi X, Li W, Barton MC, Dent SYR, Kraus WL (2018). Enhancer transcription reveals subtype-specific gene expression programs controlling breast cancer pathogenesis. Genome Res.

[18] Meers MP, Adelman K, Duronio RJ, Strahl BD, McKay DJ, Matera AG (2018). Transcription start site profiling uncovers divergent transcription and enhancer-associated RNAs in Drosophila melanogaster. BMC Genomics.

[19] Mahat DB, Kwak H, Booth GT, Jonkers IH, Danko CG, Patel RK, Waters CT, Munson K, Core LJ, Lis JT (2016). Base-pair-resolution genome-wide mapping of active RNA polymerases using precision nuclear run-on (PRO-seq). Nat Protoc.

[20] Scruggs BS, Gilchrist DA, Nechaev S, Muse GW, Burkholder A, Fargo DC, Adelman K (2015). Bidirectional transcription arises from two distinct hubs of transcription factor binding and active chromatin. Mol Cell.

[21] Samarakkody A, Abbas A, Scheidegger A, Warns J, Nnoli O, Jokinen B, Zarns K, Kubat B, Dhasarathy A, Nechaev S (2015). RNA polymerase II pausing can be retained or acquired during activation of genes involved in the epithelial to mesenchymal transition. Nucleic Acids Res.

[22] Nechaev S, Fargo DC, dos Santos G, Liu L, Gao Y, Adelman K (2010). Global analysis of short RNAs reveals widespread promoter-proximal stalling and arrest of pol II in Drosophila. Science.

[23] Rasmussen EB, Lis JT (1993). In vivo transcriptional pausing and cap formation on three Drosophila heat shock genes. Proc Natl Acad Sci U S A.

[24] Karolchik D, Baertsch R, Diekhans M, Furey TS, Hinrichs A, Lu YT, Roskin KM, Schwartz M, Sugnet CW, Thomas DJ, Weber RJ, Haussler D, Kent WJ (2003). The UCSC genome browser database. Nucleic Acids Res.

[25] Carcamo J, Buckbinder L, Reinberg D (1991). The initiator directs the assembly of a transcription factor IID-dependent transcription complex. Proc Natl Acad Sci U S A.

[26] Core LJ, Waterfall JJ, Lis JT (2008). Nascent RNA sequencing reveals widespread pausing and divergent initiation at human promoters. Science.

[27] Seila AC, Calabrese JM, Levine SS, Yeo GW, Rahl PB, Flynn RA, Young RA, Sharp PA (2008). Divergent transcription from active promoters. Science.

[28] Preker P, Nielsen J, Kammler S, Lykke-Andersen S, Christensen MS, Mapendano CK, Schierup MH, Jensen TH (2008). RNA exosome depletion reveals transcription upstream of active human promoters. Science.

[29] He Y, Vogelstein B, Velculescu VE, Papadopoulos N, Kinzler KW (2008). The antisense transcriptomes of human cells. Science.

[30] Flynn RA, Almada AE, Zamudio JR, Sharp PA (2011). Antisense RNA polymerase II divergent transcripts are P-TEFb dependent and substrates for the RNA exosome. Proc Natl Acad Sci U S A.

[31] Tome JM, Tippens ND, Lis JT (2018). Single-molecule nascent RNA sequencing identifies regulatory domain architecture at promoters and enhancers. Nat Genet.

[32] Nechaev S, Adelman K (2011). Pol II waiting in the starting gates: regulating the transition from transcription initiation into productive elongation. Biochim Biophys Acta.

[33] Liu X, Kraus WL, Bai X (2015). Ready, pause, go: regulation of RNA polymerase II pausing and release by cellular signaling pathways. Trends Biochem Sci.

[34] Gilchrist DA, Dos Santos G, Fargo DC, Xie B, Gao Y, Li L, Adelman K (2010). Pausing of RNA polymerase II disrupts DNA-specified nucleosome organization to enable precise gene regulation. Cell.

[35] Day DS, Zhang B, Stevens SM, Ferrari F, Larschan EN, Park PJ, Pu WT (2016). Comprehensive analysis of promoter-proximal RNA polymerase II pausing across mammalian cell types. Genome Biol.

[36] Gilmour DS, Lis JT (1986). RNA polymerase II interacts with the promoter region of the noninduced hsp70 gene in Drosophila melanogaster cells. Mol Cell Biol.

[37] Rougvie AE, Lis JT (1988). The RNA polymerase II molecule at the 5′ end of the uninduced hsp70 gene of D. melanogaster is transcriptionally engaged. Cell.

[38] Giardina C, Pérez-Riba M, Lis JT (1992). Promoter melting and TFIID complexes on Drosophila genes in vivo. Genes Dev.

[39] Rasmussen EB, Lis JT (1995). Short transcripts of the ternary complex provide insight into RNA polymerase II elongational pausing. J Mol Biol.

[40] Danko CG, Hah N, Luo X, Martins AL, Core L, Lis JT, Siepel A, Kraus WL (2013). Signaling pathways differentially affect RNA polymerase II initiation, pausing, and elongation rate in cells. Mol Cell.

[41] Hendrix DA, Hong JW, Zeitlinger J, Rokhsar DS, Levine MS (2008). Promoter elements associated with RNA pol II stalling in the Drosophila embryo. Proc Natl Acad Sci U S A.

[42] Juven-Gershon T, Kadonaga JT (2010). Regulation of gene expression via the core promoter and the basal transcriptional machinery. Dev Biol.

[43] Kwak H, Fuda NJ, Core LJ, Lis JT (2013). Precise maps of RNA polymerase reveal how promoters direct initiation and pausing. Science.

[44] Pertea M, Pertea GM, Antonescu CM, Chang TC, Mendell JT, Salzberg SL (2015). StringTie enables improved reconstruction of a transcriptome from RNA-seq reads. Nat Biotechnol.

[45] Sahraeian SME, Mohiyuddin M, Sebra R, Tilgner H, Afshar PT, Au KF, Bani Asadi N, Gerstein MB, Wong WH, Snyder MP, Schadt E, Lam HYK (2017). Gaining comprehensive biological insight into the transcriptome by performing a broad-spectrum RNA-seq analysis. Nat Commun.

[46] Gao Z, Lee P, Stafford JM, von Schimmelmann M, Schaefer A, Reinberg D (2014). An AUTS2-Polycomb complex activates gene expression in the CNS. Nature.

[47] Heinz S, Benner C, Spann N, Bertolino E, Lin YC, Laslo P, Cheng JX, Murre C, Singh H, Glass CK (2010). Simple combinations of lineage-determining transcription factors prime cis-regulatory elements required for macrophage and B cell identities. Mol Cell.

[48] Young RS, Kumar Y, Bickmore WA, Taylor MS (2017). Bidirectional transcription initiation marks accessible chromatin and is not specific to enhancers. Genome Biol.

[49] Meng H, Bartholomew B (2018). Emerging roles of transcriptional enhancers in chromatin looping and promoter-proximal pausing of RNA polymerase II. J Biol Chem.

[50] Andersson R, Gebhard C, Miguel-Escalada I, Hoof I, Bornholdt J, Boyd M, Chen Y, Zhao X, Schmidl C, Suzuki T, Ntini E, Arner E, Valen E, Li K, Schwarzfischer L, Glatz D, Raithel J, Lilje B, Rapin N, Bagger FO, Jørgensen M, Andersen PR, Bertin N, Rackham O, Burroughs AM, Baillie JK, Ishizu Y, Shimizu Y, Furuhata E, Maeda S, Negishi Y, Mungall CJ, Meehan TF, Lassmann T, Itoh M, Kawaji H, Kondo N, Kawai J, Lennartsson A, Daub CO, Heutink P, Hume DA, Jensen TH, Suzuki H, Hayashizaki Y, Müller F, FANTOM C, Forrest AR, Carninci P, Rehli M, Sandelin A (2014). An atlas of active enhancers across human cell types and tissues. Nature.

[51] Jimeno-González S, Ceballos-Chávez M, Reyes JC (2015). A positioned +1 nucleosome enhances promoter-proximal pausing. Nucleic Acids Res.

[52] Mavrich TN, Jiang C, Ioshikhes IP, Li X, Venters BJ, Zanton SJ, Tomsho LP, Qi J, Glaser RL, Schuster SC, Gilmour DS, Albert I, Pugh BF (2008). Nucleosome organization in the Drosophila genome. Nature.

[53] Cook PR (2010). A model for all genomes: the role of transcription factories. J Mol Biol.

[54] Feuerborn A, Cook PR (2015). Why the activity of a gene depends on its neighbors. Trends Genet.

[55] Scheidegger A, Nechaev S (2016). RNA polymerase II pausing as a context-dependent reader of the genome. Biochem Cell Biol.

[56] Yamaguchi Y, Takagi T, Wada T, Yano K, Furuya A, Sugimoto S, Hasegawa J, Handa H (1999). NELF, a multisubunit complex containing RD, cooperates with DSIF to repress RNA polymerase II elongation. Cell.

[57] Wada T, Takagi T, Yamaguchi Y, Ferdous A, Imai T, Hirose S, Sugimoto S, Yano K, Hartzog GA, Winston F, Buratowski S, Handa H (1998). DSIF, a novel transcription elongation factor that regulates RNA polymerase II processivity, is composed of human Spt4 and Spt5 homologs. Genes Dev.

[58] Rahl PB, Lin CY, Seila AC, Flynn RA, McCuine S, Burge CB, Sharp PA, Young RA (2010). C-Myc regulates transcriptional pause release. Cell.

[59] Missra A, Gilmour DS (2010). Interactions between DSIF (DRB sensitivity inducing factor), NELF (negative elongation factor), and the Drosophila RNA polymerase II transcription elongation complex. Proc Natl Acad Sci U S A.

[60] Lu H, Xue Y, Yu GK, Arias C, Lin J, Fong S, Faure M, Weisburd B, Ji X, Mercier A, Sutton J, Luo K, Gao Z, Zhou Q (2015). Compensatory induction of MYC expression by sustained CDK9 inhibition via a BRD4-dependent mechanism. Elife.

[61] Pagano JM, Kwak H, Waters CT, Sprouse RO, White BS, Ozer A, Szeto K, Shalloway D, Craighead HG, Lis JT (2014). Defining NELF-E RNA binding in HIV-1 and promoter-proximal pause regions. PLoS Genet.

[62] Vos SM, Pöllmann D, Caizzi L, Hofmann KB, Rombaut P, Zimniak T, Herzog F, Cramer P. Architecture and RNA binding of the human negative elongation factor. eLife. 2016;5:e14981. 10.7554/eLife.14981 PMC4940160 27282391

[63] Li J, Liu Y, Rhee HS, Ghosh SK, Bai L, Pugh BF, Gilmour DS (2013). Kinetic competition between elongation rate and binding of NELF controls promoter-proximal pausing. Mol Cell.

[64] Sheridan RM, Fong N, D’Alessandro A, Bentley DL (2019). Widespread backtracking by RNA pol II is a major effector of gene activation, 5′ pause release, termination, and transcription elongation rate. Mol Cell.

[65] Shlyueva D, Stampfel G, Stark A (2014). Transcriptional enhancers: from properties to genome-wide predictions. Nat Rev Genet.

[66] Gao T, He B, Liu S, Zhu H, Tan K, Qian J (2016). EnhancerAtlas: a resource for enhancer annotation and analysis in 105 human cell/tissue types. Bioinformatics.

[67] Fishilevich S, Nudel R, Rappaport N, Hadar R, Plaschkes I, Iny Stein T, Rosen N, Kohn A, Twik M, Safran M, Lancet D, Cohen D. GeneHancer: genome-wide integration of enhancers and target genes in GeneCards. Database (Oxford). 2017;2017. 10.1093/database/bax028 PMC5467550 28605766

[68] Liao Y, Smyth GK, Shi W (2014). featureCounts: an efficient general purpose program for assigning sequence reads to genomic features. Bioinformatics.

[69] Love MI, Huber W, Anders S (2014). Moderated estimation of fold change and dispersion for RNA-seq data with DESeq2. Genome Biol.

[70] Ramírez F, Ryan DP, Grüning B, Bhardwaj V, Kilpert F, Richter AS, Heyne S, Dündar F, Manke T (2016). deepTools2: a next generation web server for deep-sequencing data analysis. Nucleic Acids Res.

