# Supplementary material for: Genome-wide RNA pol II initiation and pausing in neural progenitors of the rat
Source: BMC Genomics. 2019 Jun 11;20:477. doi: 10.1186/s12864-019-5829-4 (PMC6558777; doi:10.1186/s12864-019-5829-4)
Supplement: Supplementary file 1 — Supplemental figures and supplemental figure legends. (DOCX 1323 kb) [file 12864_2019_5829_MOESM1_ESM.docx]

**SUPPLEMENTAL MATERIAL.**

**Supplemental Figure S1.** **Preparation of start-seq RNA libraries.** A. Size selection of trizol-extracted total RNA on a 15-% UREA/TBE gel (Novex/Invitrogen). The gel was stained with ethidium bromide and the region indicated with brackets was cut to extract RNA. The single-stranded RNA ladder (NEB) is loaded as size reference. B. Final scRNA library. The non-denaturing 6% TBE gel (Novex/Invitrogen) is shown. Double-stranded 25-bp DNA ladder (Invitrogen) is loaded alongside for reference. The 125-bp band corresponding to empty linker ligations is shown and was avoided from the library that was cut and purified from this ethidium bromide stained gel as indicated with brackets.

**Supplemental Figure S2. Examples of genes with high TSS-RNA levels.** Two independent replicates are shown on browser shots separately, both for 5’-ends and 3’-ends. Genes were chosen for their relevance to neural development [10]. *Fgf2* is an example of a gene with broad initiation pattern.

**Supplemental figure S3. TSS-RNA peak location positions around RefSeq-annotated gene TSS.** The graph shows frequency distribution of peak positions. Vertical bars denote +/-1SD interval after fitting of these data to normal distribution to determine genes with the “discrepancy” between annotated and TSS RNA-defined start sites within that interval that we took for reannotation.

**Supplemental Figure S4**. **DNA sequence context of gene TSSs continues for genes with lowest Start-seq signal.** Genes were separated into quartiles and Weblogo analysis was done for each quartile for RefSeq and TSS-RNA defined gene start sites.

**Supplemental Figure S5.** Metaplot analysis showing length of TSS-RNAs versus location of their 5’-ends relative to the TSS. Mean value of RNA length is plotted. Vertical lines indicate the positions of TSSs for each replicate.

**Supplemental Figure S6. RNA lengths distribution from paired end TSS-RNA reads**. RNA lengths were calculated based on paired-end sequencing of TSS-RNA libraries on a miSeq instrument, with 3,196,138 unique paired-end alignments to rn6 genome for the shown replicate. Top – TSS-RNA lengths around known gene promoters (+/-150nt from the TSS). Bottom – TSS RNA from divergent transcription initiation, collected from an interval from -50 to -300nt on the strand opposite to gene TSSs.

­

**Supplemental Figure S7. RNA sequencing of new genes. Left panel.** FPKM values were obtained using feature counts, averaged for two biological replicates of RNA-seq. normalized to gene exon length for depth of sequencing coverage. The bar graph shows median with range. **Right panel.** Metagene plot of 5’-positions of TSS-RNA around new gene TSSs.
